# Fibre density and fibre-bundle cross-section of the corticospinal tract are distinctly linked to psychosis-specific symptoms in antipsychotic-naïve patients with first-episode schizophrenia

**DOI:** 10.1007/s00406-023-01598-7

**Published:** 2023-04-04

**Authors:** Tina D. Kristensen, Jayachandra M. Raghava, Martin W. Skjerbæk, Thijs Dhollander, Warda Syeda, Karen S. Ambrosen, Kirsten B. Bojesen, Mette Ø. Nielsen, Christos Pantelis, Birte Y. Glenthøj, Bjørn H. Ebdrup

**Affiliations:** 1 Center for Neuropsychiatric Schizophrenia Research and Center for Clinical Intervention and Neuropsychiatric Schizophrenia Research, Mental Health Centre Glostrup, Copenhagen University Hospital, Nordstjernevej 41, 2600 Glostrup, Denmark; 2 Functional Imaging Unit, Department of Clinical Physiology, Nuclear Medicine and PET, Rigshospitalet, Glostrup, Denmark; 3 Developmental Imaging, Murdoch Children’s Research Institute, Victoria, Australia; 4 Melbourne Neuropsychiatry Centre, Department of Psychiatry, University of Melbourne and Melbourne Health, Victoria, Australia; 5 Department of Clinical Medicine, Faculty of Health and Medical Sciences, University of Copenhagen, Copenhagen, Denmark

**Keywords:** Fixel-based analysis, White matter, First-episode antipsychotic-naïve schizophrenia, Substance use, Psychopathology

## Abstract

Multiple lines of research support the dysconnectivity hypothesis of schizophrenia. However, findings on white matter (WM) alterations in patients with schizophrenia are widespread and non-specific. Confounding factors from magnetic resonance image (MRI) processing, clinical diversity, antipsychotic exposure, and substance use may underlie some of the variability. By application of refined methodology and careful sampling, we rectified common confounders investigating WM and symptom correlates in a sample of strictly antipsychotic-naïve first-episode patients with schizophrenia. Eighty-six patients and 112 matched controls underwent diffusion MRI. Using fixel-based analysis (FBA), we extracted fibre-specific measures such as fibre density and fibre-bundle cross-section. Group differences on fixel-wise measures were examined with multivariate general linear modelling. Psychopathology was assessed with the Positive and Negative Syndrome Scale. We separately tested multivariate correlations between fixel-wise measures and predefined psychosis-specific versus anxio-depressive symptoms. Results were corrected for multiple comparisons. Patients displayed reduced fibre density in the body of corpus callosum and in the middle cerebellar peduncle. Fibre density and fibre-bundle cross-section of the corticospinal tract were positively correlated with suspiciousness/persecution, and negatively correlated with delusions. Fibre-bundle cross-section of isthmus of corpus callosum and hallucinatory behaviour were negatively correlated. Fibre density and fibre-bundle cross-section of genu and splenium of corpus callosum were negative correlated with anxio-depressive symptoms. FBA revealed fibre-specific properties of WM abnormalities in patients and differentiated associations between WM and psychosis-specific versus anxio-depressive symptoms. Our findings encourage an itemised approach to investigate the relationship between WM microstructure and clinical symptoms in patients with schizophrenia.

**Supplementary Information:**

The online version contains supplementary material available at 10.1007/s00406-023-01598-7.

## Introduction

Abnormalities in neuronal connectivity of the brain are implicated in the pathophysiology of schizophrenia [1]. In particular, the dysconnectivity hypothesis implies that microstructural alterations of white matter (WM) contribute to the symptom expression in patients [2]. Although WM alterations have been demonstrated in several meta-analyses [3, 4], findings are widespread [5] and non-specific for patients with schizophrenia [6]. This variability may partly be explained by multiple confounders in magnetic resonance imaging (MRI) studies [7], such as crossing fibres and partial volume effects from grey matter (GM) and cerebrospinal fluid (CSF), along with clinical heterogeneity, illness duration, antipsychotic medication, and substance use. Studies carefully designed to rectify the effect of such confounders are central for the understanding of the neurobiological underpinnings of schizophrenia, aiming to advance their diagnostic and prognostic specificity [8].

Diffusion weighted imaging (DWI) is a non-invasive MRI imaging technique and diffusion tensor imaging (DTI) is a specific physical model [9, 10] that has been extensively applied for in vivo investigation of WM microstructure [11]. However, DTI-derived measures such as fractional anisotropy (FA) are non-specific, and the model cannot resolve the fibre orientation, presence of crossing fibres, and partial volume effects from adjacent GM and CSF featuring across the brain at the conventional spatial resolution of the DWI data [12].

**Fig. 1:**
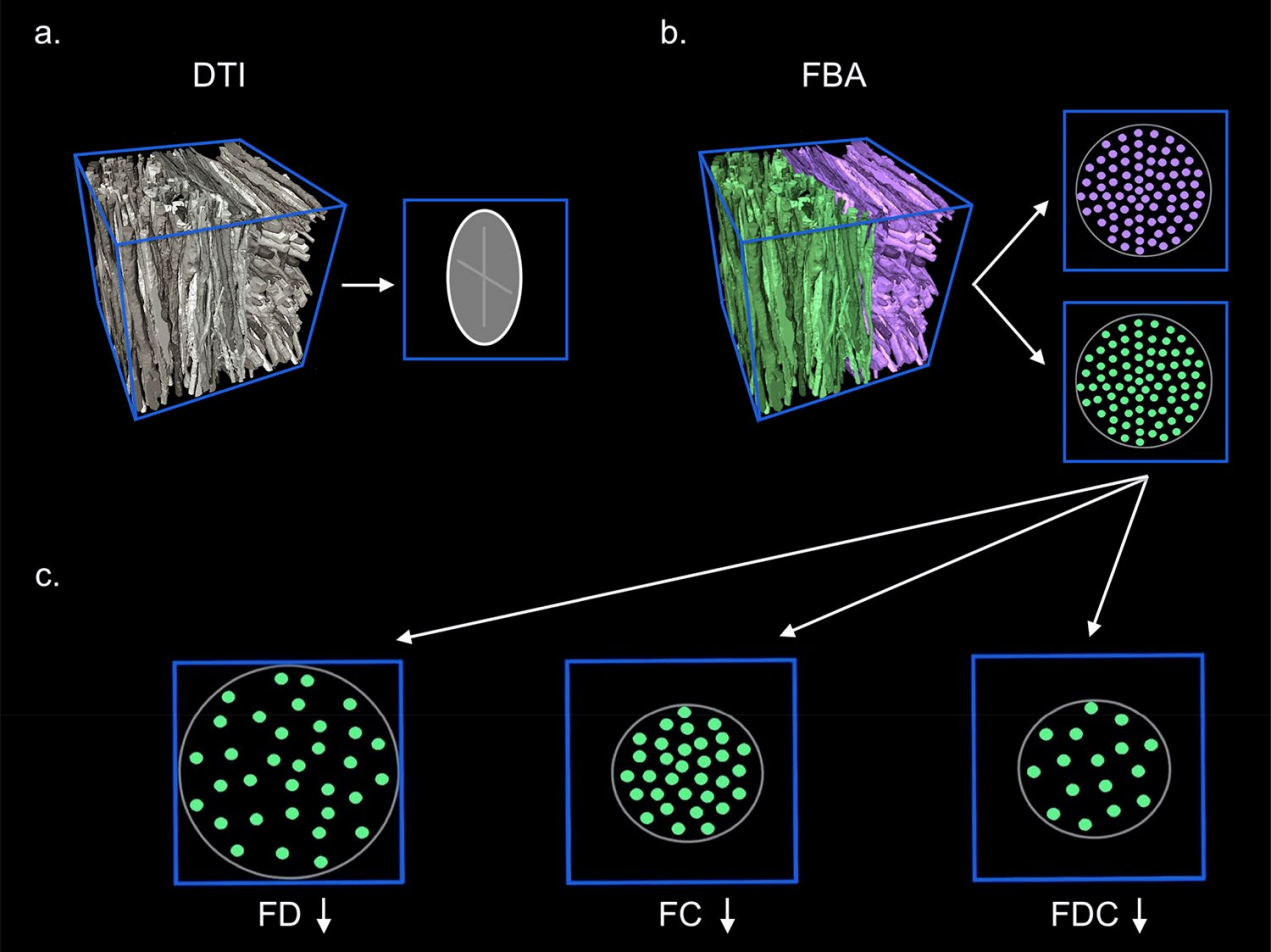
Fixel-based analyses. Figure 1 illustrates the top row **a** Diffusion tensor imaging (DTI) provides general information about the diffusion signal within a voxel (blue box). The measures derived are averaged to one single principal direction of axonal fibres (demonstrated by the elongated oval), which is affected by crossing fibre populations. **b** Using spherical deconvolution, the fixel-based analyses (FBA) enable identification of the individual fibre populations with different orientations (purple and green axons) within the voxel. Quantifiable measures are extracted for each individual fibre population. **c** The bottom row illustrates the fixel-based measures of fibre density (FD), fibre-bundle cross-section (FC), and the combined measure of fibre density and fibre-bundle cross-section (FDC). Reduced FD reflects a decrease in the microscopical intra-cellular volume of axons within a voxel. Reduced FC indicates a macroscopic decrease of the cross-sectional size of a particular fibre bundle

Fixel-based analysis (FBA) is a recently developed framework that addresses the limitations of the DTI model, allowing for assessment of more biological specific micro- and macrostructural measures of WM [13, 14]. The term fixel refers to each individual fibre population within a voxel. In a typical FBA, fixels are derived from WM fibre orientation distributions (FODs), which are computed using constrained spherical deconvolution (CSD) techniques [14]. A method called single-shell 3-tissue CSD (SS3T-CSD) was recently proposed [15] to obtain similar results compared to multi-shell multi-tissue CSD (MSMT-CSD) [16], using only single-shell DWI data to reliably model the tissue compartments of WM, grey matter and cerebrospinal fluid [17]. Thus, the FBA-derived measure of apparent fibre density (FD) captures microstructural information that is proportional to the total intra-axonal volume of a specific fibre population within a voxel. At the macrostructural level, FBA provides a morphological measure reflecting the total cross-sectional size of an individual fibre bundle (FC) [13]. Finally, the measure of fibre density and fibre-bundle cross-section (FDC) combines the properties of FD and FC, providing a measure sensitive to both intra-axonal volume and the cross-sectional size of a specific fibre bundle[16] (see Fig. 1 for illustration). Reductions in fixel-wise measures appear to reflect neurodegenerative processes [18, 19] which, in conjunction with a strong neurodevelopmental aethiology [20, 21], have been suggested as part of the pathophysiology of schizophrenia [22, 23].

A recent extensive review reports a steep increase in FBA studies between 2018 and 2021, including both healthy as well as a range of clinical populations [14]. Apparent advantages over the non-specific measures derived from DTI-modelling are demonstrated thoroughly even on single-shell data, as fixel-wise measures appear largely insensitive to signal related to myelin and other non-WM cells, fluids and tissue[14]. Furthermore, several of the reviewed FBA studies demonstrated larger effect sizes and higher sensitivity to group differences compared to DTI-modelling, particularly in WM regions featuring crossing fibres [24–26]. To our knowledge, only two studies have employed FBA in patients with schizophrenia: Stämpfli et al. (2019) [27] investigated FD and FA alterations in 20 clinically stable and medicated patients with schizophrenia or schizoaffective disorder, and reported reduced FD in widespread WM tracts in patients compared to healthy controls, but no group difference on FA. Additionally, a negative correlation between FD of the thalamic radiation (TR) and positive symptoms were reported, but it was highly associated with current antipsychotic dose. Although the authors suggest that FD may be a promising approach to detect subtle WM alterations and potential clinical associations, the small and diagnostic heterogenous sample of medicated patients with 10-year mean duration of illness calls for further studies in larger samples of patients not exposed to clinical confounders.

Grazioplene et al. (2018) [28] performed FBA as well as DTI on a larger group of 54 first-episode and 27 chronic patients with schizophrenia. They reported that clusters where patients displayed lower FA compared to HC overlapped substantially with WM regions containing complex fibre architecture, and that FA was strongly associated with crossing fibres complexity. Using FBA, patients displayed lower FD in corpus callosum (CC) compared to HC. However, no associations with specific clinical symptoms were reported from this heterogenous patient sample. Although both studies on patients with schizophrenia reported FD alterations with an apparent higher specificity of fixel-wise measures compared to FA, clinical implications remain unclear due to the medicated and heterogenous patient groups. Furthermore, neither study investigated the measures of FC and FDC, and common confounders to the FBA studies may have contributed to the findings [29].

The Positive and Negative Syndrome Scale (PANSS) [30] is one of the most used measures of symptom severity in patients with schizophrenia. DTI studies have identified symptomatic correlates to global and regional WM, commonly applying a total score comprising the sum of positive, negative, and general symptom domains, or the respective domain scores [31, 32]. However, the psychometric properties of PANSS have been questioned [33–35] and several subscales have been proposed [36, 37] to achieve a more cardinal description with a higher degree of specificity [38, 39]. Hence, we a priori applied selected items from two distinct, predefined symptom domains in order to identify potential WM alterations related to either psychosis-specific symptoms central to schizophrenia [40], or symptoms representing more general and unspecific psychopathology [41] (see Methods for details).

Based on the aforementioned FBA studies [27, 28], we predicted that patients would present with reduced FD in widespread WM regions including CC, when compared to HCs. Further, we expected a negative correlation between fixel-wise measures and PANSS items.

Next, we investigated regional specificity of potential correlations between fixel-wise measures and the psychosis-specific symptoms and anxio-depressive symptoms, respectively. Finally, we explored if recreational substance use confounded the identified group differences and clinical correlates.

## Methods

Data were acquired as part of two consecutive cohorts PECANS 1 and 2 (Pan European Collaboration on Antipsychotic Naïve Schizophrenia) conducted in the Capital Region of Copenhagen, Denmark, in the period of 2008–2019 [42, 43]. The studies comply with the Committee on Biomedical Research Ethics (H-D-2008-088, H-3-2013-149) and with the Helsinki Declaration of 1975, and were registered at Clinicaltrials.gov (NCT01154829, NCT02339844). All participants provided oral and written informed consent prior to inclusion in the study.

### Participants

Patients aged 18–45 years were recruited from psychiatric hospitals and outpatient clinics. Diagnoses were confirmed using the Schedules for Clinical Assessment in Neuropsychiatry (SCAN), version 2.1[44]. Exclusion criteria for patients were previous treatment with antipsychotics or methylphenidate, a current diagnosis of drug dependency according to the International Classification of Diseases 10^th^ Edition (ICD-10), treatment with antidepressant medication within the last month, involuntary admission or treatment, or severe physical illness. Previous substance abuse, and current occasional substance use and benzodiazepine prescriptions were accepted. HCs were recruited using online advertising and matched to patients based on age, sex, and parental socioeconomic status. Exclusion criteria for HCs were any physical or mental illness, having a first degree relative with psychotic symptoms, or substance abuse during the last three months before inclusion. All participants reported quantity and frequency of their substance use (alcohol, tobacco, cannabis, stimulants, hallucinogens, opioids, other illicit drugs) and provided a urine drug-screen test (Rapid Response, Jepsen HealthCare, Tune, DK). A neuroradiologist examined all MRI scans, and participants with overt MRI pathology were excluded.

In the current study, we restricted analyses to patients with an ICD-10 diagnosis of schizophrenia (DF20.x) to increase sample homogeneity. From our sample of 109 antipsychotic-naïve first-episode psychosis patients pooled from the 2 cohorts (PECANS I and II), we included 86 patients and 112 HCs for the primary analysis. For sensitivity analyses regarding the potential confounding effect of substance use, we applied the same rigorous criteria as in a previous DTI-study [45] and excluded participants with a lifetime regular use (i.e. ‘once or a few times per week’) of one or more recreational drugs (cannabis, stimulants, hallucinogens, opioids, or other illicit drugs), resulting in a substance-free subsample of 62 patients and 104 healthy controls. Supplementary Figure S1 displays details of the inclusion process.

### Assessments

#### Clinical assessments

Trained raters assessed psychopathology using the PANSS [46]. For correlation analyses we separately tested items from two symptom domains based on previous studies: Lefort-Besnard et al. (2018) [40] applied machine learning to examine underlying patterns of PANSS symptom profiles and reported the 3 PANSS items P1 (delusions), P3 (hallucinatory behaviour) and P6 (suspiciousness/persecution) to be the most prominent positive symptoms showing a consistent heavy load across distinct schizophrenia subtypes [40]. Furthermore, P1, P3, and P6 represent central ‘pathognomonic’ symptoms of schizophrenia, as reflected in strong correlations with overall illness severity [47], sensitivity to improvements following antipsychotic treatment [37], and a sound prediction of relapse [48]. Yazaji et al. (2002) [41] performed a principal component analyses on all 30 PANSS items and proposed an anxio-depressive symptom domain comprising the general psychopathology items of G1 (somatic concern), G2 (anxiety), G3 (guilt feelings), and G6 (depression) [41], also previously applied in a study on patients with schizophrenia [49].

The Global Assessment of Functioning (GAF) scale [50] was used to assess level of functioning in patients.

#### Image acquisition

MRI data were acquired as described in our previous DTI studies on subgroups of the current sample [45, 51, 52]. Briefly, we used a Philips Achieva 3.0 T MRI scanner (Philips Healthcare, Best, The Netherlands) with a 32-channel SENSE Head Coil (Invivo, Orlando, Florida, USA). Volumes of whole-brain diffusion weighted images were acquired in 30 non-collinear directions (*b* = 1000 s/mm^2^) and 5 non-diffusion weighted images (*b* = 0 s/mm^2^). See Supplementary Text S2 for details.

#### Image processing

In this work, we followed the recommended steps of FBA [53]. Both sets of dMRI data were denoised using an overcomplete Local Principal Component Analysis (PCA) method [54] in MATLAB software (version 2017b, Natick, Massachusetts: The MathWorks Inc.). The FMRIB diffusion toolbox (part of FSL 6.0.0) [55, 56] and MRtrix3Tissue (https://3tissue.github.io) [57] were used for pre-processing, starting with bias correction [57], followed by correction for eddy current-induced susceptibility distortions and subject movements [58, 59]. Head motion parameters were recorded (3 translational and 3 rotational motion parameters). Next, the data were spatially up-sampled to 1.3 × 1.3 × 1.3 mm^3^ resolution using cubic b-spline interpolation [57]. Whole brain masks were generated. For each subject, the 3-tissue response functions were estimated for single-fibre WM, GM and CSF using an unsupervised method [60, 61]. A group-level response function was calculated by averaging the response functions of all the subjects for each tissue type. Using these group average response functions, Single-Shell 3-Tissue constrained spherical deconvolution (SS3T-CSD) was performed for each subject to obtain WM-like fibre orientation distribution (FOD) maps [60]. We then applied multi-tissue informed intensity normalisation in the log-domain with added gradual outlier rejection [62]. A study-specific population template was generated with iterative registration and averaging FOD maps from 40 subjects (20 patients and 20 controls matched on age, sex, and parental socio-economic status). Each subject’s WM FOD was non-linearly registered to the population template [63, 64]. These FODs were segmented to produce fibre-specific fixels and were reoriented according to the former nonlinear transformation to the template space [13, 65]. For each subject, FD, FC, and FDC across all WM fixels were computed. Finally, we performed a whole-brain probabilistic tractography on the FOD template which serves to quantify fixel-to-fixel connectivity for the purpose of fixel-wise smoothing and connectivity-based fixel enhancement [24]. Structural T1 weighted images were processed using Freesurfer (version 7.1.1) software. Sequence Adaptive Multimodal Segmentation (SAMSEG) tool was used to calculate intracranial volumes (ICV) for all the subjects [66].

MRI quality control was performed by visually inspecting all dMRI residual maps to identify artefactual signal intensities [67, 68]. Thirteen subjects that did not pass the QA were excluded. Three quality metrics (temporal signal-to-noise ratio (TSNR), maximum voxel intensity outlier count (MAXVOX), and mean voxel intensity outlier count (MEANVOX)) were computed from each dMRI volume using a quality assessment method previously described in Roalf et al. [69]. The quality metrics in the present study ranged between the ‘good’ and ‘excellent’ (Supplementary Table S3).

### Statistical analyses

Analyses of descriptive data were performed using SPSS version 25.0 (Armonk, NY, US). Distributions of continuous data were tested for normality by visual inspection of histograms, outliers were examined, and Levene’s Test for Equality of Variances was applied. Group differences were tested using Pearson’s χ^2^ test, Fisher’s Exact Test, the Mann–Whitney *U* test, or analysis of variance (ANOVA) as appropriate.

Fixel-based analyses were performed using connectivity-based fixel enhancement and non-parametric permutation testing with 5000 permutations [24]. Primary analyses of group comparisons were performed using Multivariate General Linear Model (GLM) with measures of FD, FC, and FDC. Age, sex, cohort, ICV, and six motion parameters were used as covariates of no-interest.

Secondary analyses comprised tests of partial correlation between fixel-wise measures (FD, FC, and FDC) as independent variables in two separate multivariate models, including the 3 psychosis-specific and the 4 anxio-depressive symptoms, respectively. First, we modelled fixel-wise measures as independent variables (predictors) and item P1, P3, and P6 as dependent variables (outcome), along with age, sex, cohort, ICV, and six motion parameters as covariates in one model. Second, we identically modelled fixel-wise measures as predictors, and items G1, G2, G3, and G6 as outcomes, along with identical covariates in one model. By including all the domain-specific items in one multivariate model, we aimed to identify the variance associated with each symptom which is orthogonal to (not overlapping with) the remaining symptoms included.

Sensitivity analyses comprised identical tests of group differences and correlations in the substance-free subsample. Furthermore, we post hoc tested identical models on group differences, comparing patients and controls on DTI-derived measures of fractional anisotropy (FA) in the full sample, as well as in the substance-free sample (See Supplementary text S4 for details of the processing pipeline). Finally, we tested correlations between PANSS items of interest (P1, P3, P6, G1, G2, G3, G6, G16).

The anatomic locations and size of the clusters comprising significant fixels were extracted using the John Hopkins University (JHU) white matter tractography atlas after transforming the significant fixels from the template space to standard Montreal Neurological Institute (MNI) space [70, 71]. The WM bundles to which significant fixel clusters belonged to were validated using TractSeg software [72]. Fixel wise measures are relative and have arbitrary units. Hence, the mean percentage effect size (%r) within the significant clusters were estimated in order to express the effect size relative to controls for the group differences. Similarly, mean beta coefficients (β) within the significant clusters were reported for single group correlation analyses. All significant results are reported after corrections for multiple comparisons using family-wise error (FWE) with a threshold of *p* < 0.05. In post hoc tests we additionally corrected for multiplicity using the false discovery rate (FDR) [73].

## Results

Demographic characteristics of patients and HCs are reported in Table 1. The match on age, gender, parental socioeconomic status, and handedness was balanced. There were a few patients with lifetime alcohol abuse or dependency, but regular use of alcohol was more common among the HCs (*p* = 0.019). Additionally, patients used more nicotine, cannabis, benzodiazepines, opioids, stimulants, hallucinogens, and other illicit drugs. Substance-free patients did not differ significantly from the full sample of patients on any demographic and clinical variables (Supplementary Table S5 and S6). Clinical data on patients are reported in Table 2. Patients had significantly lower premorbid IQ (DART,* p* < 0.010).

**Table 1:** Sociodemographic data full sample

Variable mean (SD)/Percent (*N*)	Patients (*N* = 86)	Controls (*N* = 112)	Significance group effect
Age years, mean (SD)	25 (6)	24 (5)	*p* = 0.45, *F* = 0.93
Gender			*p* = 0.97, ***χ***^2^ < 0.01
Male	49% (42)	49% (55)	
Female	51% (44)	51% (57)	
Parental SES			*p* = 0.45, *χ*^2^ = 1.60
Low	18% (15)	13% (14)	
Medium	54% (45)	53% (59)	
High	28% (23)	34% (38)	
Handedness			*p* = 0.28, *χ*^2^ = 2.55
Right	85% (72)	85% (91)	
Left	7% (6)	11% (12)	
Ambidextrous	8% (7)	4% (4)	
DART, mean (SD)	**19 (8)**	**22 (6)**	***p* < 0.01, *F* = 12.16**
Alcohol			***p* < 0.01, *χ***^**2**^** = 24.05**
Dependency	1% (1)	0% (0)	
Harmful use	6% (5)	0% (0)	
Use regularly	61% (51)	89% (97)	
Tried few times	29% (24)	9% (10)	
Never tried	4% (3)	2% (2)	
Tobacco			***p* < 0.01, *χ***^**2**^** = 22.41**
Dependency	8% (7)	1% (1)	
Harmful use	1% (1)	2% (2)	
Use regularly	37% (32)	16% (17)	
Tried few times	15% (13)	32% (35)	
Never tried	38% (33)	50% (53)	
Cannabis			***p* < 0.01, *χ***^**2**^** = 17.53**
Dependency	1% (1)	0% (0)	
Harmful use	7% (6)	0% (0)	
Use regularly	17% 15)	6% (7)	
Tried few times	45% (39)	50% (54)	
Never tried	29% (25)	44% (47)	
Benzodiazepines			***p* < 0.01, *χ***^**2**^** = 35.30**
Dependency	0% (0)	0% (0)	
Harmful use	0% (0)	0% (0)	
Use regularly	4% (3)	0% (0)	
Tried few times	27% (22)	1% (1)	
Never tried	69% (56)	99% (105)	
Opioids			***p* < 0.01, *χ***^**2**^** = 15.39**
Dependency	0% (0)	0% (0)	
Harmful use	0% (0)	0% (0)	
Use regularly	1% (1)	0% (0)	
Tried few times	19% (16)	3% (3)	
Never tried	80% (69)	97% (104)	
Stimulants			***p* < 0.01, *χ***^**2**^** = 13.24**
Dependency	0% (0)	0% (0)	
Harmful use	0% (0)	0% (0)	
Use regularly	6% (5)	0% (0)	
Tried few times	24% (20)	11% (12)	
Never tried	70% (59)	89% (94)	
Hallucinogens			***p* < 0.01, *χ***^**2**^** = 7.64**
Dependency	0% (0)	0% (0)	
Harmful use	0% (0)	0% (0)	
Use regularly	0% (0)	0% (0)	
Tried few times	15% (12)	4% (4)	
Never tried	85% (68)	96% (102)	
Other drugs			***p* = 0.034, *χ***^**2**^** = 6.77**
Dependency	0% (0)	0% (0)	
Harmful use	0% (0)	0% (0)	
Use regularly	2% (1)	0% (0)	
Tried few times	10% (6)	2% (2)	
Never tried	89% (54)	98% (98)	
MRI-Translational motion^a^, mean (SD)			
Tx	0.858 (0.237)	0.8721 (0.188)	*p* = 0.645
Ty	− 0.402 (0.247)	− 0.3757 (0.240)	*p* = 0.452
Tz	− 0.015 (0.700)	0.0327 (0.474)	*p* = 0.571
MRI-Rotational motion^a^, mean (SD)			
Rx	5.03E-05 (0.009)	− 5.96E-04 (0.008)	*p* = 0.604
Ry	− 0.001 (0.004)	− 5.43E-04 (0.004)	*p* = 0.416
Rz	− 0.002 (0.005)	− 0.0017 (0.004)	*p* = 0.941

Significant effect of group is marked in bold

^a^ Between group effect calculated with 2 sample *t* test

*N* number, *SES* socio-economic status, *SD* standard deviation

**Table 2:** Clinical data on patients with schizophrenia

Variable mean (s.d.)	Full sample (*n* = 86)	Substance-free sample (*n* = 62)	Substance-using sample (*n* = 24)	Group effect substance-free vs. Substance-using sample
GAF	36.7 (7.5)	34.4 (13.5)	33.2 (11.2)	*p* = 0.70, *F* = 0.15
DUI/Weeks	74.9 (82.6)	60.5 (66.1)	102.1 (103.8)	*p* = 0.08, *F* = 3.10
DUP/Weeks	101.9 (132.3)	103.8 (129.2)	91.0 (165.0)	*p* = 0.85, *F* = 0.04
DART	18.9 (8.0)	19.5 (8.0)	17.2 (8.4)	*p* = 0.27, *F* = 1.22
P1 Delusions	3.9 (1.1)	3.9 (1.2)	3.9 (0.8)	*p* = 0.94, *F* < 0.01
P3 Hallucinations	3.4 (1.5)	3.4 (1.5)	3.5 (1.4)	*p* = 0.66, *F* = 0.19
P6 Suspiciousness/persecution	3.6 (1.4)	3.6 (1.4)	3.6 (1.3)	*p* = 0.86, *F* = 0.03
G1 somatic concern	2.2 (1.6)	2.3 (1.6)	2.1 (1.6)	*p* = 0.62, *F* = 0.25
G2 Anxiety	3.9 (1.5)	3.9 (1.3)	3.9 (1.8)	*p* = 0.86, *F* = 0.03
G3 guilt feelings	2.7 (1.5)	2.6 (1.5)	2.9 (1.6)	*p* = 0.43, *F* = 0.62
G6 depression	3.5 (1.3)	3.5 (1.1)	3.3 (1.5)	*p* = 0.59, *F* = 0.29
PANSS positive total	19.4 (4.0)	19.2 (4.1)	20.1 (3.8)	*p* = 0.35, *F* = 0.88
PANSS negative total	20.2 (6.9)	20.0 (6.7)	20.6 (7.5)	*p* = 0.72, *F* = 0.13
PANSS general total	39.7 (8.6)	39.2 (9.0)	40.8 (7.6)	*p* = 0.43, *F* = 0.62
PANSS total score	79.2 (16.1)	78.4 (16.1)	81.5 (14.3)	*p* = 0.42, *F* = 0.66

*DART* Danish version of the National Adult Reading Test, *DUI* duration of illness, *DUP* duration of untreated psychosis, *GAF* The Global Assessment of Functioning, *N* number, *PANSS* Positive And Negative Syndrome Scale, *SD* standard deviation;

### Group differences of fixel-wise measures

**Fig. 2:**
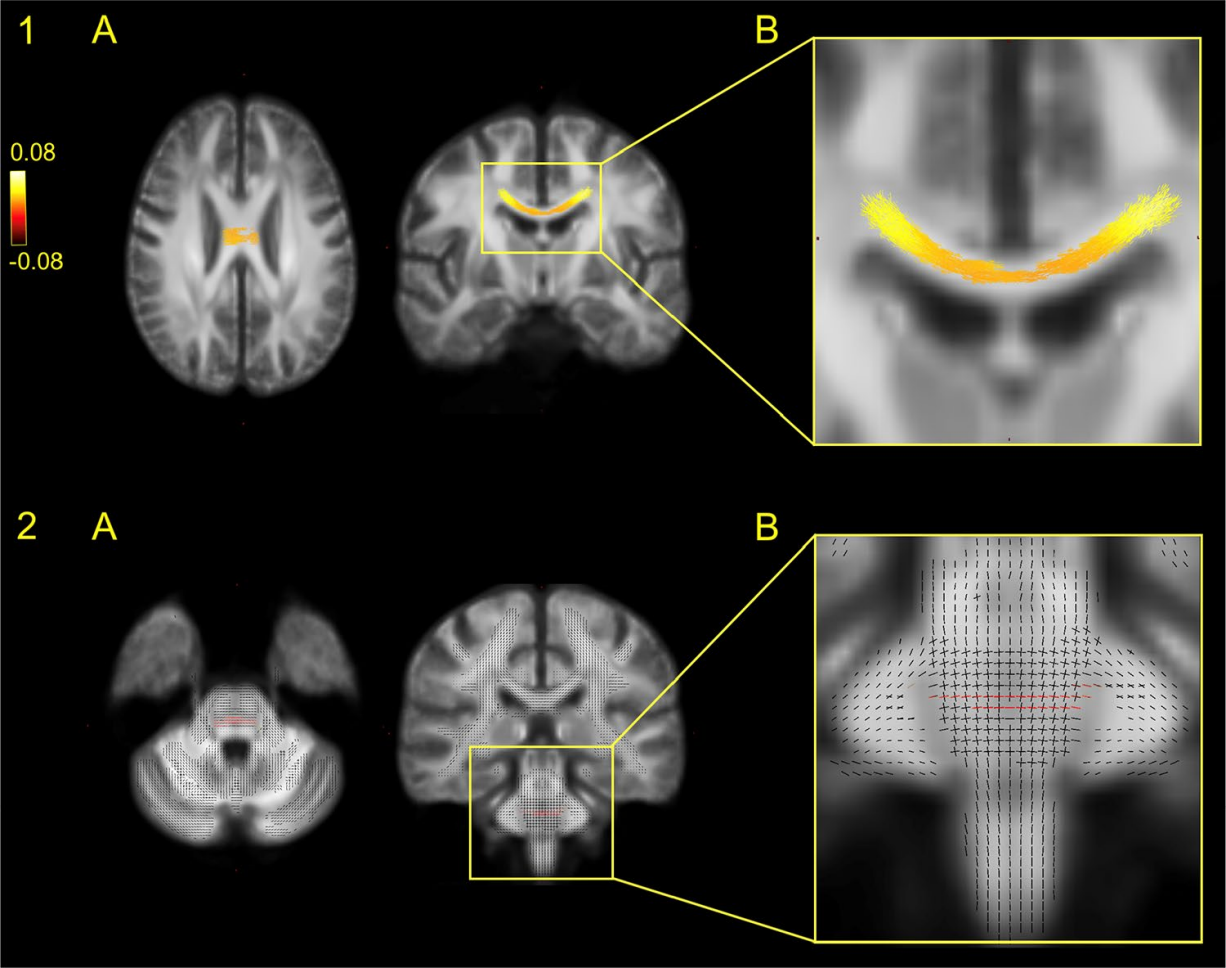
Group difference on fibre density. Figure 2 illustrates the location of the main group difference on FD comparing patients with schizophrenia with healthy controls. Group comparisons were performed using Multivariate General Linear Model (GLM). Family-wise error (FWE) with a threshold of *p* < 0.05 was applied to correct for multiple comparisons. Top row 1A is the significant group difference in the body of corpus callosum in an axial and coronal plane, comparing the full sample of patients with schizophrenia with healthy controls. The cluster is coloured yellow–red according to effect size of the group difference, enhanced in image 1B. Bottom row 2A is the significant group difference in middle cerebellar peduncle in an axial and coronal plane, comparing the subsample of patients and controls with no recreational substance use. Significant fixels are coloured red, while non-significant fixels are black, enabling the identification of crossing fibres, enhanced in image 2B

In the full sample, we found reduced FD in patients in a widespread cluster of the body of corpus callosum (CC) (*%r* = 3.503) and of the left middle cerebellar peduncle (MCP) (*p* < 0.05, Fig. 2). In the substance-free sample, we found reduced FC of the MCP bilaterally. (*p* < 0.05, *%r* = 0.077). In post hoc test of FD in CC, comparing the full patient sample to the substance-free sample, we found no group difference (*p* = 0.72).

### Correlations between fixel-wise measures and psychosis-specific symptoms

Significant results are reported in Table 3. For details on smaller significant clusters and the specific localizations (X, Y, Z coordinates), see Supplementary Table S7 and Figure S8.

**Table 3:** Correlations between fixel-wise measures and psychopathology

PANSS item	Fixel measure	Correlation	Cluster size (voxels)	Beta coefficient	White matter tract
PANSS Psychosis-specific items					
Full sample 86 patients					
P6 (Suspiciousness/persecution)	FC FDC	Positive Positive	5909 3205	*β* = 0.030 *β* = 0.035	CST, bilaterally
Substance-free subsample 62 patients					
P1 (Delusions)	FDC	Negative	392	*β* = − 0.060	Left CST
P3 (Hallucinatory behaviour)	FC	Negative	172	*β* = − 0.036	Isthmus of CC
P6 (Suspiciousness/persecution)	FC FDC	Positive Positive	10,295 3870	*β* = 0.035 *β* = 0.042	CST, bilaterally
PANSS Anxio-depressive items					
Full sample 86 patients					
G1 (Somatic concern)	FC	Negative	2446	*β* = − 0.034	Splenium of CC
G3 (Guilt feelings)	FD	Negative	154	*β* = − 0.011	Genu of CC
Substance-free subsample 62 patients					
G1 (Somatic concern)	FC FDC	Negative Negative	1436 2129	*β* = − 0.042 *β* = − 0.066	Splenium of CC
G3 (Guilt feelings)	FD	Negative	68	*β* = − 0.016	Genu of CC

Correlation tests were performed using Multivariate General Linear Model (GLM). Family-wise error (FWE) with a threshold of *p* < 0.05 were applied to correct for multiple comparisons. Only significant associations and the largest cluster within the white matter tracts are reported, details on smaller clusters within the same tract and specific localizations (X, Y, Z coordinates) in Supplementary Table S7

*β* Absolute mean beta coefficient, *FC* fibre-bundle cross-section, *FD* fibre density, *FDC* fibre density and cross-section, *G* general; *P* positive, *PANSS* Positive And Negative Syndrome Scale

**Fig. 3:**
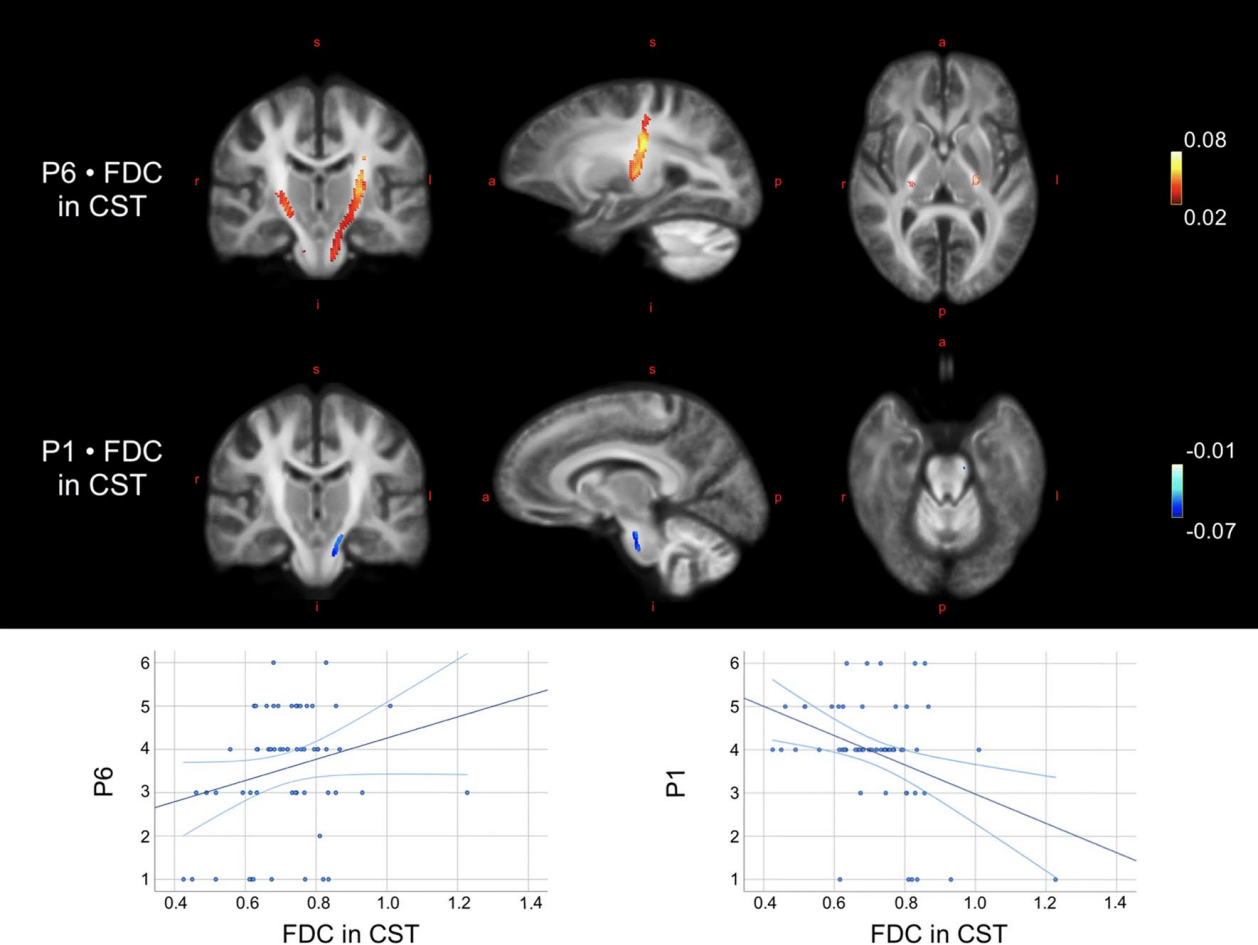
Correlations between fixel-wise measures and psychosis-specific symptoms. Top image illustrates the localization of the significant correlations between fixel-wise measures and the PANSS items P1 and P6 in corticospinal tract (CST) in the substance-free sample. Blue colours indicate negative correlations, red/yellow colours indicate positive correlations. Colour is graded according to beta estimate as proxy to effect size. Images are displayed in radiological directions. Bottom row: scatterplots illustrate the associations between FDC of the significant fixels of CST and the items scores of P1 and P6, respectively. *a* anterior, *FDC* fibre density and cross-section, *i* inferior, *l* left, *p* posterior, *r* right, *s* superior

In the full sample, FC (*β* = 0.030) and FDC (*β* = 0.035) of the corticospinal tract (bilaterally) correlated positively with PANSS item P6 (suspiciousness/persecution) (*p* < 0.05), likewise in the substance-free sample, FC (*β* = 0.035) and FDC (*β* = 0.042). Additionally, FDC of the left corticospinal tract correlated negatively with PANSS item P1 (delusions) (*β* = − 0.060) (Fig. 3 visualises clusters and mean beta coefficients). FC of the isthmus of CC correlated negatively with PANSS item P3 (hallucinatory behaviour) (*β* = − 0.036, *p* < 0.05).

### Correlations between fixel-wise measures and anxio-depressive symptoms

In the full sample, FC of the splenium of CC correlated negatively with PANSS item G1 (somatic concern) (*β* = − 0.034); and FD of the genu of CC correlated negatively with PANSS item G3 (guilt feelings) (*β* = − 0.011, *p* < 0.05, Supplementary Figure S7).

Similarly in the substance-free sample, FC (*β* = − 0.042) and FDC (*β* = − 0.066) of the splenium of CC correlated negatively with PANSS item G1 (somatic concern); and FD of the genu of CC correlated negatively with PANSS item G3 (guilt feelings) (*β* = − 0.016, *p* < 0.05).

### Post hoc* examinations*

The DTI-analyses on FA in the full and the substance-free sample did not reveal any significant group differences when comparing patients to controls (Supplementary Text S3).

Non-parametric correlation tests between PANSS items revealed after FDR correction for multiplicity positive correlations between P6 and P1 in the full sample, as well as between P6 and G1 in the substance-free sample. Finally, our explanatory test of associations between P1, P6 and G16 (active social avoidance) revealed that G16 was highly significant positively correlated with P6 (*r* = 0.413, *p* = 0.001), but not P1 after correction for multiplicity.

## Discussion

In this study, we identified group differences in WM and revealed associations between fixel-wise measures and two distinct domains of psychopathology in differentially located WM tracts.

We confirmed the expected reduction of FD in a large cluster of the body of CC in the full sample of patients compared to HCs, corroborating the findings reported by Grazioplene et al. (2018) [28] on medically stabilised chronic patients. Although they demonstrated an association between illness duration and FD in CC, our results indicate that callosal alterations are present from illness onset and independent of antipsychotic medication. Lower FD is proportional to reduced intra-axonal volume (i.e. reduced axonal matter), which here may indicate aberrant structural underpinnings for the interhemispheric information transfer across the body of CC in patients. The finding is corresponding to previous WM studies in patients with schizophrenia, reporting reduced volumen and lower FA in particularly the midsagittal segment of CC [74].

We did not find reduced FD in widespread WM regions as expected but identified a smaller area in the left MCP where patients displayed lower FD than HCs. Notably, in the substance-free sample we found reduced FC in even larger and bilateral clusters of MCP. MCP contains afferent fibres connecting pons to the cerebellum, and is a major projection pathway relaying sensory information from the cerebral cortex to cerebellum [75]. The reduction of the cross-sectional size of the MCP fibre bundle may represent a pathophysiological feature specific for schizophrenia, whereas the more widespread findings reported by Stämpfli et al. [27] may to some extend be explained by effects of chronicity, antipsychotic medication, and recreational substance use. The location of MCP would be in accordance with the cognitive dysmetria theory by Andreasen [76], proposing that dysconnectivity in specifically the cortico-cerebellar networks underlays the disturbed sensory information processing in patients with schizophrenia, potentially explaining positive psychotic symptoms [77, 78]. However, as both cluster- and effect size were small, results must be interpreted modestly.

The current study is to our knowledge the first to demonstrate the link between distinct domains of psychopathology and specific locations of WM alterations using FBA. Our main clinical finding was the positive correlation between FC and FDC of the CST and suspiciousness/persecution, which was replicated in a larger cluster in the substance-free sample. Additionally, delusions were negatively correlated with FDC more ventrally in the left CST in the substance-free sample.

The CST is a major projection tract, originating from the motor cortex and descending into the spinal cord via the midbrain, and is considered a vital pathway for the modulation of both motor and somatosensory information in the cortico-cerebellar network [79]. Our findings in CST may at the first glance be surprising, considering that formations of psychotic symptoms in schizophrenia have primarily been linked to alterations in the dopamine system [80–82]. Studies using functional MRI have identified three major fronto-striatal-thalamic dopaminergic networks [83]. The mesostriatal and particularly the mesolimbic pathways have been investigated as to understand the dopaminergic disturbances in schizophrenia [84–86], and alterations in reward and incentive salience systems have been identified [87]. In line with these findings, previous studies have reported links between delusions, paranoia and aberrant white matter in mesolimbic and -striatal tracts, such as the supero-lateral medial forebrain bundle and the inferior fronto-occipital fasciculus [88, 89]. The dopaminergic systems are also anatomically connected to the motor cortex via ascending fibres originating in substantia nigra (the nigrostriatal pathway) [90]. However, CST is mainly composed of descending pyramidal cells using glutamate as excitatory neurotransmitter [91], suggested to play a role in the cortical excitatory to inhibitory imbalance in schizophrenia [92]. Although the complex interplay between neuronal circuits regulated by neurotransmitters is not well understood [93], a recent review by Howes and Shatalina (2022) [94] suggests a model of schizophrenia integrating the dopamine hypothesis and the role of cortical excitation-inhibition imbalance. Indeed, dysconnectivity of motor pathways has been linked to prediction error and mismatch negativity in functional MRI studies in patients with schizophrenia [95, 96]. We speculate if the positive correlation, i.e. more suspiciousness/persecution associated with larger CST fibre bundles and more intra-axonal volume could reflect this hypo- and hyper-connectivity, contributing differently to specific clinical symptoms in schizophrenia [97].

Additionally, CST has been demonstrated to be a direct amygdala-motor pathway involved in functional coactivation of amygdala and motor-related areas during perception of emotional expressions [98], and corticospinal downstream projections have been shown to be involved in a motor system reaction to fearful expressions [99]. Functional hyperactivity in amygdala have been demonstrated in patients with schizophrenia when in a paranoid state, compared to nonparanoid patients and controls [100, 101]. One study linked severity of paranoia in patients with schizophrenia with increased functional connectivity within the core limbic circuit and prefrontal cortex, reflecting amplified threat processing and impaired emotion regulation [102]. According to the integrated model, synaptic abnormalities (caused by neurodevelopmental features, including stress) disrupts the excitation/inhibition balance and could lead to disinhibition of excitatory cortical projections regulating the mesostriatal dopamine neurons, further resulting in dopamine dysregulation and psychotic symptoms [94]. Studies have reported positive correlations between fixel-wise measures and resting state- as well as task-based functional activity [103, 104], but as our data do not allow for such conclusions, future multimodal investigations of associations between fixel-wise measures and functional activity linked to clinical symptoms is necessary.

The direction of the correlations between psychopathology and fixel-wise measures were as expected all negative, except for item P6 (suspiciousness/persecution). Intriguingly, the correlations between FDC of the CST and delusions and suspiciousness/persecution were opposite, i.e. less delusions, but more suspiciousness/persecution were associated with larger CST fibre bundles and more intra-axonal volume. We speculated that the opposite correlations partly may be a consequence of our multivariate models identifying the aspects of P6 which are not overlapping with P1, i.e. it is not the delusional, but rather specifically the emotional / interpersonal aspect of P6 which is positively associated with fibre density and fibre-bundle cross-section of CST. We post hoc confirmed this emotional / interpersonal representation, as P6 contrary to P1 was strongly associated with G16 (active social avoidance). Indeed, CST has been denoted as a major structural underpinning of an emotional-motor- [105] and mirror-neuron system [106, 107] linked to the processing and interpretation of emotional states, actions and intentions of others [108, 109], which is known to be impaired in patients with schizophrenia [110, 111]. However, the more ventrally located subregion of the correlation to delusions may be a component in these opposite correlations. A recent review state dopamine signalling in more dorsal regions of the striatum to be particularly associated with threat-related stimuli [112], and it has been suggested that neurochemical mechanisms differ according to subregions on a ventral–dorsal axis, indicating that hypoconnectivity appears more prominent in ventrally located regions, contrary to hyper-connectivity in dorsal localizations [113].

P3 (hallucinatory behaviour) was negative correlated with FC in the isthmus of CC. WM fibres traversing the isthmus connects the superior temporal and inferior parietal GM regions bilaterally [114, 115], and have in DTI studies been associated with persistent [116] and auditory hallucinations [97, 117]. Nonetheless, our findings in CC mainly indicate neuropathological mechanisms non-specific for psychosis, as the correlations between fixel-wise measures and the anxio-depressive symptoms all were located to segments of the CC. Hence, fibre density and fibre-bundle cross-section of callosal WM appear associated with hallucinations as well as anxio-depressive symptoms, although in different segments. Alterations of callosal WM as a cross-diagnostic commonality for mental disorders such as schizophrenia, bipolar disorder [118, 119], and major depressive disorder [120] are in accordance with DTI studies demonstrating lower FA of CC in patients compared to controls. The negative correlation between FD located to the genu of CC and G3 (guilt feelings) appears to correspond to findings from one recent FBA study, revealing reduced FC and FDC of the genu of CC in patients with major depressive disorder [121].

The direction of all correlations between psychopathology and fixel-wise measures located to the CC were all negative as expected, suggesting that reduced callosal fibre-bundle cross-section and less intra-axonal volume were associated with more severe psychopathology. However, to validate specific structural underpinnings of the symptom domains, the findings should be replicated in studies ideally including patients with non-psychotic disorders as contrast.

A major strength of this study is the large sample size of antipsychotic-naïve patients with first-episode schizophrenia, which enabled sensitivity analyses of a substance-free sample. Although our sensitivity analyses comparing the substance-free patients to HCs did not reveal group differences in CC, the post hoc tests indicated that the lack of significant group differences in CC could be attributed to loss of power, rather than a potential effect of recreational substance use. Hence, we have meticulously controlled for the effects of common confounders as we aimed for.

Some limitations should be considered. Our results from a refined group of antipsychotic-naïve patients with no lifetime recreational substance use represent limited external validity, as patients with schizophrenia commonly present both medicated and with substance use in clinical settings. However, the internal validity is robust considering the clinically homogenous sample.

Finally, our diffusion data were acquired using the single-shell, with a relatively low b-value of 1000 s/mm^2^ and 30 gradient directions, which might compromise the specificity to intra-axonal signal. Nonetheless studies using SSMT-CSD technique have shown that single-shell DWI data produces similar results results compared to multi-shell DWI data [17]. Additionally, the biological accuracy of single-shell data processed with CSD has been confirmed in post-mortem histological studies [122]. Hence, we believe that the biological interpretation of our results is reliable and convey biologically relevant findings, even though they rely on less optimal data acquisition parameters.

Concluding, our study reveals fibre-specific characteristics of WM alterations in antipsychotic-naive patients with first-episode schizophrenia, suggesting these alterations play a central role in both positive and general psychopathology in differentiated symptom-specific WM tracts. Particularly, fibre characteristics of CST appeared related to positive psychotic symptoms in a refined sample of patients without any recreational substance use. Overall, FBA appears to demonstrate an advance in specificity when examining the neuronal underpinnings of central psychotic symptoms and generalised psychopathology.

### Supplementary Information

Below is the link to the electronic supplementary material.

Supplementary file1 (DOCX 427 KB)

## Data Availability

Data sharing not applicable to this article as no datasets were generated or analysed during the current study.
