# Supplementary material for: Fibre density and fibre-bundle cross-section of the corticospinal tract are distinctly linked to psychosis-specific symptoms in antipsychotic-naïve patients with first-episode schizophrenia
Source: Eur Arch Psychiatry Clin Neurosci. 2023 Apr 4;273(8):1797–812. doi: 10.1007/s00406-023-01598-7 (PMC10713712; doi:10.1007/s00406-023-01598-7)
Supplement: Supplementary file 1 — Supplementary file1 (DOCX 427 KB) [file 406_2023_1598_MOESM1_ESM.docx]

**Figure S1 Flow chart**

**Text S2 Image acquisition**

MRI data were acquired using a Philips Achieva 3.0 T whole body MRI scanner (Philips Healthcare, Best, The Netherlands) with a 32-channel SENSE Head Coil (Invivo, Orlando, Florida, USA). Thirty volumes of whole-brain diffusion weighted images were acquired in 30 non-collinear directions (b = 1000 s/mm^2^) and 5 non-diffusion weighted images (b = 0 s/mm^2^) using single shot spin-echo echo-planar imaging. Acquisition of all volumes were repeated with opposite phase encoding direction to enable correction for susceptibility distortions using the following parameters: acquisition matrix = 128 × 99, field of view (FOV) = 240 × 240 mm^2^, number of slices = 75, slice thickness = 2.0 mm (no gap), reconstructed voxel dimensions = 1.88 × 1.88 × 2 mm^3^, TR/TE = 7035/ 68 ms, parallel imaging SENSE factor = 3(AP), flip angle = 90°, total scan duration = 8 min 42 sec.

High-resolution three-dimensional T1-weighted images were acquired using sagittal slices and the following parameters: acquisition matrix = 304 × 299, field of view (FOV) = 240 × 240 mm^2^, number of slices = 200, slice thickness = 0.8 mm, reconstructed voxel dimensions = 0.75 × 0.75 × 0.80 mm^3^, TR/TE = 10/4.6 ms, inversion time = 964 ms, flip angle = 8^o^, total scan duration = 10.01 minutes.

**Table S3. Image quality metrics**

|  | **TSNR**  **Mean (SD)** | **MAXVOX**  **Mean (SD)** | **MEANVOX**  **Mean (SD)** |
| --- | --- | --- | --- |
| Patients and HCs in the full sample (N=198) | 9.86  (1.09) | 6631  (10240) | 819.11  (687.21) |
| Roalf et al. (N=147)  “Poor” | 5.52  (0.93) | 14497  (8667) | 2001.50  (1080.20) |
| Roalf et al. (N=468)  “Good” | 6.9  (0.68) | 7165  (7189) | 830.40  (597.10) |
| Roalf et al. (N=742)  “Excellent” | 7.37  (0.55) | 1684  (1741) | 378  (164.10) |

*Table S3 shows mean (SD) of quality metrics on DWI data for the full sample of antipsychotic-naïve patients with first episode schizophrenia and healthy controls, as well as for the quality assessment groups defined by Roalf et al. Three image quality metrics (temporal signal-to-noise ratio (TSNR), maximum voxel intensity outlier count (MAXVOX) and mean voxel intensity outlier count (MEANVOX)) were calculated from each subjects DW image. The measured quality metrics in this study ranged between the ‘good’ and ‘excellent’ quality.*

*Abbreviations: DWI: diffusion weighted imaging; SD: standard deviation*

**Text S4** Post hoc DTI analyses of fractional anisotropy (FA)

**Image acquisition was identical of the FBA.**

**Image processing:**

The two sets of diffusion weighted scans (acquired in opposite phase-encoding directions) were denoised to reduce random rician noise with overcomplete Local PCA method (Manjón et al. 2013) using MATLAB software (version 2014a). FMRIB diffusion toolbox (part of FSL 5.0.9) (Jenkinson et al. 2012; Smith et al. 2004) was used for further pre-processing, starting with eddy current correction and head motion correction (Jenkinson et al. 2002), followed by a correction of susceptibility distortions (Andersson et al. 2003). For head motion, 3 translational and 3 rotational motion parameters were recorded. Next, data were resampled to 0.93 × 0.93 × 1mm3 resolution using non-local MRI upsampling (Manjón et al. 2010). Non-brain tissue was removed with brain extraction tool (BET) (Smith 2002). FA were calculated using DTIFIT (Jenkinson et al. 2012). FA, data were transformed to standard space and skeletonised using the standard tract- based spatial statistics (TBSS) pipeline (Smith et al. 2006). First, FA data were non-linearly registered using FNIRT (Andersson et al. 2007) onto the standard FMRIB58 FA tem- plate. Next, the nearest maximum FA values of each registered FA image were projected onto a WM skeleton derived from the FMRIB58 template and thresholded at FA > 0.2.

**Table S5. Sociodemographic data comparing healthy controls to the substance-free subsample**

| Variable  Mean (S.D.) / Percent (N) | Patients  (N=62) | controls  (N=104) | Significance  Group effect |
| --- | --- | --- | --- |
| Age mean (SD) | 25 (6) | 24 (5) | *p*=0.33, F=0.95 |
| Gender |  |  | *p*=0.52, χ^2^=0.42 |
| Male | 42 % (26) | 47 % (49) |  |
| Female | 58 % (36) | 53 % (55) |  |
| Parental SES |  |  | *p*=0.91, χ^2^=0.19 |
| Low | 15 % (9) | 13 % (13) |  |
| Medium | 52 % (31) | 53 % (55) |  |
| High | 33 % (20) | 34 % (35) |  |
| Handedness |  |  | *p*=0.12, χ^2^=4.20 |
| Right | 82 % (51) | 84 % (84) |  |
| Left | 7 % (4) | 12 % (12) |  |
| Ambidextrous | 11 % (7) | 4 % (4) |  |
| DART | **19.5 (8)** | **22.4 (6)** | ***p*<0.01, F=7.00** |

*Table S5*

*Significant effect of group is marked in bold.*

*Abbreviations: DART: Danish version of the National Adult Reading Test; DUI: duration of illness; DUP: duration of untreated psychosis; GAF: The Global Assessment of Functioning; N: number; SES: socio-economic status; SD: standard deviation;*

**Table S6.**

**Sociodemographic data comparing the full patient sample to the substance-free subsample**

| Variable  Mean (S.D.) / Percent (N) | Patients  (N=86) | Substance-free patients  (N=62) | Group effect  comparing NoU vs. substance using patients (N=24) |
| --- | --- | --- | --- |
| Age mean (SD) | 25 (6) | 25 (6) | *p*=0.69, F=0.16 |
| Gender |  |  | *p*=0.04, χ^2^=4.24 |
| Male | 49 % (42) | 42 % (26) |  |
| Female | 51 % (44) | 58 % (36) |  |
| Parental SES |  |  | *p*=0.15, χ^2^=3.86 |
| Low | 18 % (15) | 15 % (9) |  |
| Medium | 54 % (45) | 52 % (31) |  |
| High | 28 % (23) | 33 % (20) |  |
| Handedness |  |  | *p*=0.24, χ^2^=2.88 |
| Right | 85 % (72) | 82 % (51) |  |
| Left | 7 % (6) | 7 % (4) |  |
| Ambidextrous | 8 % (7) | 11 % (7) |  |

*Table S6*

*Abbreviations: N: number; SES: socio-economic status; SD: standard deviation*

*The significant difference on gender when comparing substance using to* substance-free *patients were 33.3% (N=8) females and 66.7% (N=16) males in the substance using patients versus 58.1% (N=44) females and 41.9% (N=42) males in the* substance-free *patients.*

**Table S7. Location and cluster-size of significant correlations**

| **PANSS item** | **Fixel measure** | **Correlation** | **Size**  **(voxels)** | **Localization**  **(X,Y,Z)** | **White matter tract** |
| --- | --- | --- | --- | --- | --- |
| **PANSS items Psychosis domain** | | | | | |
| **Full sample 86 patients** | | | | | |
| **P6**  (Suspiciousness/persecution) | FC  FDC | Positive  Positive | 5909  390  69  16  3205  29 | -3,-24,-36  19,-31,-32  21,-17,-7  -10,-36,-32  -3,-24,-36  -16,-24,56 | CST, bilaterally |
| **Substance-free subsample 62 patients** | | | | | |
| **P1**  (Delusions) | FDC | Negative | 392 | -4,-23,-36 | Left CST |
| **P3**  (Hallucinatory behavior) | FC | Negative | 172 | -12,-33,27 | Isthmus of CC |
| **P6**  (Suspiciousness/persecution) | FC  FDC | Positive  Positive | 10295  274  3870  1109  70 | -3,-24,-36  25,-22,14  -3,-24,-36  18,-20,-9  10,-23,-31 | CST, bilaterally |
| **PANSS items anxio-depressive domain** | | | | | |
| **Full sample 86 patients** | | | | | |
| **G1**  (Somatic concern) | FC | Negative | 2446 | 23,-42,4 | Splenium of CC |
| **G3**  (Guilt feelings) | FD | Negative | 154  24 | -3,23,0  11,26,0 | Genu of CC |
| **Substance-free subsample 62 patients** | | | | | |
| **G1**  (Somatic concern) | FC  FDC | Negative  Negative | 1436  275  152  2129 | 1,-39,15  15,-43,22  27,-50,15  24,-51,4 | Splenium of CC |
| **G3**  (Guilt feelings) | FD | Negative | 68 | 3,23,2 | Genu of CC |

**Figure S8. Correlations between fixel-based measures and psychopathology**


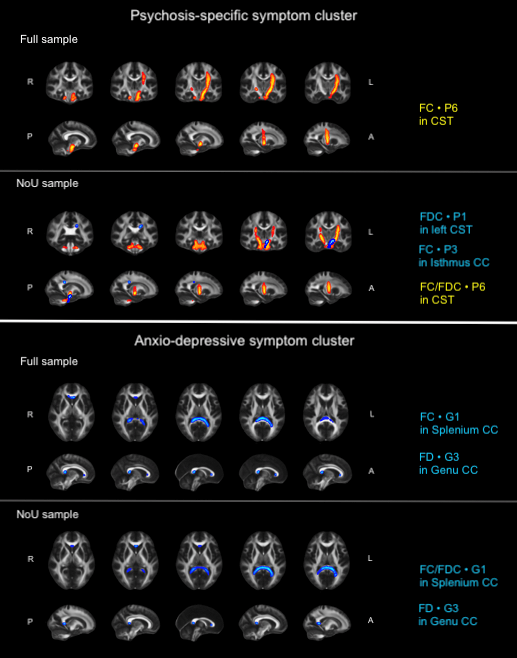


*Figure S8.* *illustrates the localization of the correlations between fibre-based measures and the PANSS-items from the two symptom-clusters, in the full sample and substance-free (NoU) sample, respectively. Blue colours indicate negative correlations, red/yellow colours indicate positive correlations. Images are displayed in radiological directions.*

*Abbreviations: A: anterior; CC: corpus callosum; CST: corticospinal tract; FC: fibre cross-section; FD: fibre density; FDC: fibre density and cross-section; L: left; P: posterior; R: right*
